# Endocannabinoid‐related compounds in gastrointestinal diseases

**DOI:** 10.1111/jcmm.13359

**Published:** 2017-10-09

**Authors:** Marcella Pesce, Alessandra D'Alessandro, Osvaldo Borrelli, Stefano Gigli, Luisa Seguella, Rosario Cuomo, Giuseppe Esposito, Giovanni Sarnelli

**Affiliations:** ^1^ Department of Clinical Medicine and Surgery ‘Federico II’ University of Naples Naples Italy; ^2^ Division of Neurogastroenterology & Motility Great Ormond Street Hospital and University of College (UCL) London UK; ^3^ Department of Physiology and Pharmacology ‘Vittorio Erspamer’ La Sapienza University of Rome Rome Italy

**Keywords:** endocannabinoid system, gastrointestinal pathophysiology, functional gastrointestinal disorders, non‐alcoholic steatohepatitis, inflammatory bowel disease

## Abstract

The endocannabinoid system (ECS) is an endogenous signalling pathway involved in the control of several gastrointestinal (GI) functions at both peripheral and central levels. In recent years, it has become apparent that the ECS is pivotal in the regulation of GI motility, secretion and sensitivity, but endocannabinoids (ECs) are also involved in the regulation of intestinal inflammation and mucosal barrier permeability, suggesting their role in the pathophysiology of both functional and organic GI disorders. Genetic studies in patients with irritable bowel syndrome (IBS) or inflammatory bowel disease have indeed shown significant associations with polymorphisms or mutation in genes encoding for cannabinoid receptor or enzyme responsible for their catabolism, respectively. Furthermore, ongoing clinical trials are testing EC agonists/antagonists in the achievement of symptomatic relief from a number of GI symptoms. Despite this evidence, there is a lack of supportive RCTs and relevant data in human beings, and hence, the possible therapeutic application of these compounds is raising ethical, political and economic concerns. More recently, the identification of several EC‐like compounds able to modulate ECS function without the typical central side effects of cannabino‐mimetics has paved the way for emerging peripherally acting drugs. This review summarizes the possible mechanisms linking the ECS to GI disorders and describes the most recent advances in the manipulation of the ECS in the treatment of GI diseases.

**Table:** 

**• Introduction**
** – Endocannabinoid‐related compounds**
** – The endocannabinoid system and the control of gastrointestinal motility**
** – The endocannabinoid system and the control of visceral sensitivity**
** – The endocannabinoid system and the control of intestinal inflammation**
**• The endocannabinoid system in gut pathophysiology**
** – The endocannabinoid system and functional dyspepsia**
** – The endocannabinoid system in irritable bowel syndrome**
** – The endocannabinoid system in inflammatory bowel disease**
** – The endocannabinoid system in liver disease**
** – Endocannabinoids in non‐alcoholic fatty liver disease**
**• Conclusions**
**• Conflict of interest**

## Introduction

**Figure 1:**
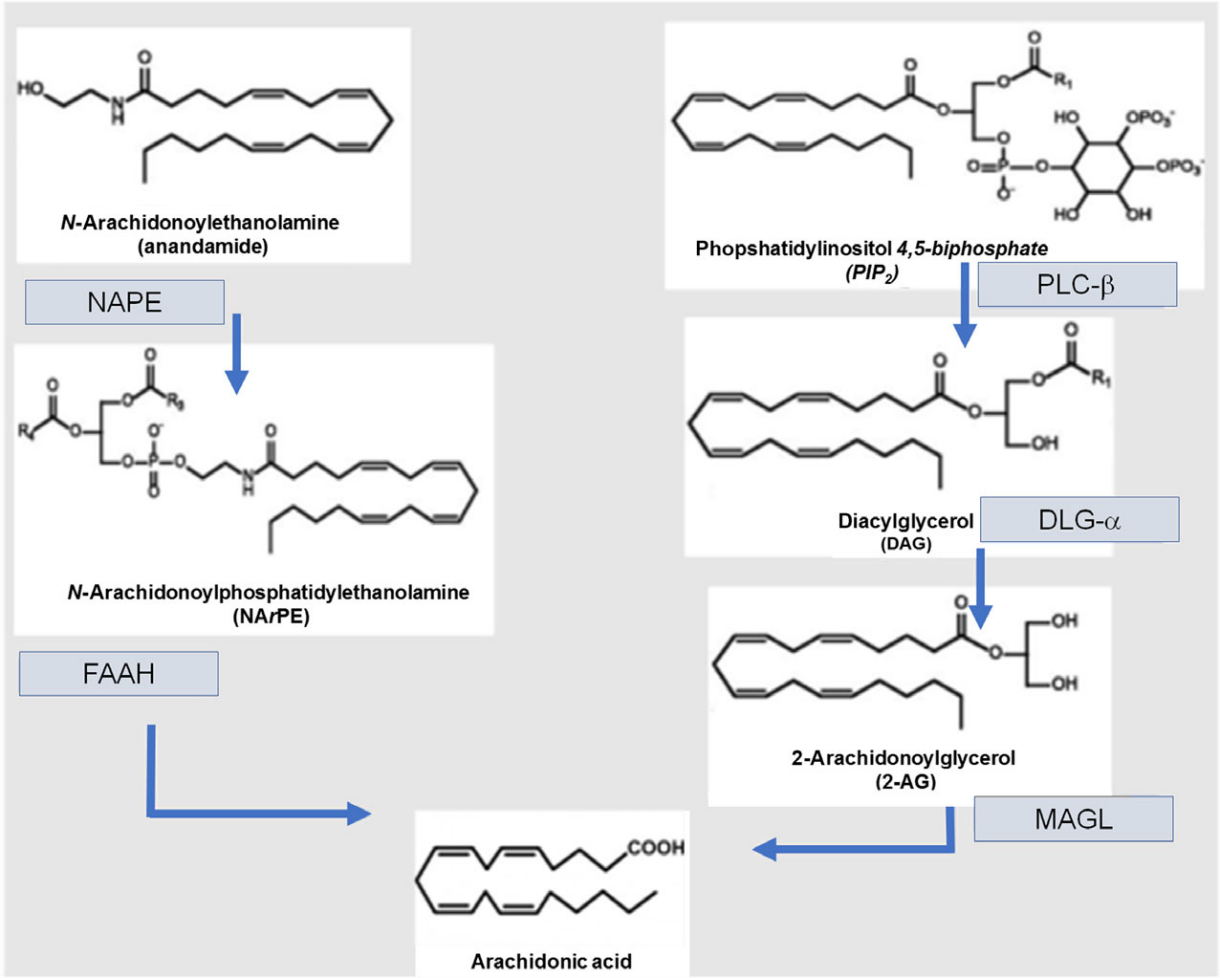
Schematic overview of the enzymes involved in EC metabolism. Anandamide (AEA) and 2‐acylglycerol (2‐AG) are the two best‐recognized stereotypical ECs. Both are synthetized by hydrolysis from membrane lipid precursors, namely *N‐*arachidonoyl‐phosphatidylethanolamine (NArPE) and phosphatidylinositol‐4,5‐bisphosphate (PIP2) for AEA and 2‐AG, respectively. Both AEA and 2‐AG, after the binding with CB receptors, are rapidly removed by membrane transporters and converted into arachidonic acid by fatty acid amide hydrolase (FAAH) and monoacylglycerol lipase (MAGL), respectively.

*Cannabis sativa* plant is the most commonly used illicit drug for recreational purposes worldwide, with estimated 16 million users in the United States 1, 2. At present, many patients use cannabis anecdotally to achieve symptomatic relief from a wide variety of symptoms, commonly of GI origin, particularly nausea and pain 3, 4, 5. The therapeutic efficacy of cannabis in the treatment of GI dysfunction relies on the fact that the GI tract is endowed with cannabinoid receptors and *N‐*arachidonoylethanolamine (anandamide, AEA) and 2‐arachidonoylglycerol (2‐AG), their best‐characterized endogenous ligands 6, 7. Together with their synthetizing and degrading enzymes, they embody the endocannabinoid system (ECS), a ubiquitous and complex system involved in the control of gut homoeostasis. Since first coined in 1995 8, the term ‘endocannabinoids’ (ECs) has been enlarged to a number of recently, yet only partially, identified endogenous ligands, such as 2‐arachidonoylglycerol ether (noladin ether), *N*‐arachidonoyl‐dopamine (NADA) and *O*‐arachidonoylethanolamine (virodhamine) 9. In recent years, several lipid‐derived mediators, closely resembling typical ECs, have been described, raising questions on the different pathophysiological role of these compounds 10, 11, 12, 13. These analogues [namely *N*‐linoleylethanolamine (LEA), *N*‐oleoylethanolamine (OEA), *N*‐palmitoylethanolamine (PEA) and *N*‐stearoylethanolamine (SEA)] are structurally related to classical ECs and have been shown to act synergistically, either enhancing the effects of prototypic ECs (the so‐called entourage effect) or displaying unique effects (seethe ‘Endocannabinoid‐related compounds’ section). An overview of the principal ECs and of the enzymes responsible for their metabolism is proposed in Figure 1. Different from other transmitters, the ECs and their congeners are not stored in intracytoplasmic vesicles, but synthetized from membrane precursors in an ‘on‐demand’ fashion 13. After their release into the extracellular space, these short‐lived compounds are rapidly removed from membrane transporters and degraded by specific enzymes (Fig. 1) 14. The ECs are able to exert their multifaceted activities by binding a large number of receptors that have not been fully identified, so far. The best‐characterized receptors are cannabinoid receptors 1 and 2 (CB1 and CB2), two G‐protein‐coupled receptors expressed in both peripheral and central nervous systems, as well as by a number of non‐neural cells 6, 15, 16. CB1 is responsible for the classical psychotropic effects of marijuana and is mainly expressed in the CNS 17. In the GI tract, CB1 is expressed in both myenteric and submucosal plexuses of the enteric nervous system (ENS), mostly by motoneurons, interneurons and primary afferent neurons but also by epithelial cells 18. Conversely, CB2 predominantly shows a peripheral distribution, with the highest rate of expression on immune cells 19, 20, but it is also found on enteric neurons 21. In rodent models, CB2 appears to be expressed by intestinal epithelial cells; however, this evidence has not been confirmed in both other animal models and human beings 22, 23, 24. As mentioned above, ECs and their related compounds exhibit several non‐CB1/CB2‐mediated effects by binding other receptors with different affinity. The orphan G‐protein‐coupled receptor 55 (GPR55), identified in 1999, has been proposed as the third CB receptor, and although it has been found in the jejunum, ileum and colon, its distribution has not been extensively studied 25. One of the best‐characterized non‐CB receptors for ECs is the transient receptor potential vanilloid type 1 (TRPV1), mainly located on the primary afferent nerve fibres 26. Originally identified as receptors for the capsaicin 27, TRPV1 receptors are known for being activated by NADA and AEA as effectively as capsaicin 28. AEA is a full agonist on TRPV1 receptors, but it also exerts indirect effects by binding CB1 29. Furthermore, a number of ECs have been shown to bind peroxisome proliferator‐activated receptors (PPARs). *In vitro* studies showed that AEA, noladin and virodhamine are receptor agonists to PPARα, while 2‐AG binds to PPARβ/δ 30. Taken together, the bewildering redundancy of the ECS and the different sites of action of the ECs account for the great variety of actions exhibited by these compounds *in vivo*.

### Endocannabinoid‐related compounds

**Figure 2:**
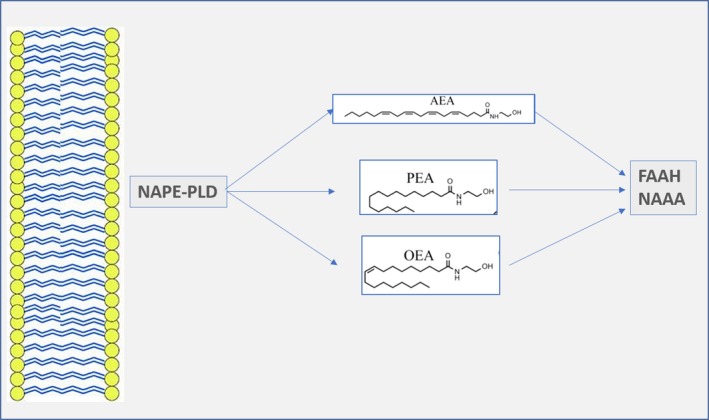
Biosynthesis and degradation of *N*‐acylethanolamides (NAEs) and possible points of interaction between AEA and its related compounds. Similar to AEA,* N*‐palmitoylethanolamine (PEA) and *N*‐oleoylethanolamine (OEA) are synthesized by *N*‐acylphosphatidylethanolamine‐specific phospholipase D (NAPE‐PLD) from membrane precursors. Unlike AEA, PEA and OEA exhibit no binding affinity on CB1/CB2 receptors, but they can enhance AEA activity at TRPV1 receptors. PEA and OEA are degraded by either fatty acid amide hydrolase (FAAH) or *N*‐acetylethanolamine‐hydrolysing acid amidase (NAAA). By competing with AEA for FAAH (mainly OEA) or by down‐regulating FAAH expression (predominantly PEA), they can increase AEA levels.

EC‐like compounds, such as *N*‐acylethanolamides (NAEs), have a close structural resemblance with classical ECs, but display no activity on CB receptors 10, 31, 32. However, these compounds share some biological activities and similar biosynthetic pathways of those of typical ECs, particularly AEA. AEA synthesis is, indeed, coupled with the formation of PEA, OEA and LEA 10, 33, 34. Although OEA and PEA do not directly activate cannabinoid receptors, they are thought to indirectly potentiate ECS signalling *via* the ‘entourage effect’ by either competing with stereotypical ECs for enzymatic degradation or increasing their receptor binding affinity 10 (Fig. 2). PEA and OEA are, indeed, both substrates of FAAH, the enzyme responsible for AEA degradation. By either competing with AEA for FAAH or inducing FAAH down‐regulation 35, 36, PEA and OEA could reduce AEA catabolism and ultimately increase AEA concentrations. Furthermore, independently of FAAH, PEA and OEA are able to enhance AEA effects at TRPV1 receptors 37, 38. OEA and PEA can activate, even if with different receptor affinity, PPARα, the G‐protein‐coupled receptor GPR119 and the TRPV1 39, 40, 41, 42. A growing body of evidence has shown that these compounds are involved in the control of a wide variety of functions, including the control of food intake 43, 44, neuroprotection 45 and inhibition of pain and inflammation 46, 47. PEA levels increase in inflamed tissues, possibly as a protective effect to exert its well‐recognized anti‐inflammatory and analgesic properties 46. In biopsies from patients with coeliac disease, levels of both PEA and AEA were increased 48. It has been shown that by selectively binding PPARα receptors, PEA is able to down‐regulate iNOS expression and nuclear factor‐κB (NFκB) activation, and in turn the inflammation in a number of chronic inflammatory conditions, including experimental and human models of inflammatory bowel disease (IBD) 49, 50, 51. PEA is indeed able to significantly inhibit the expression of S100B and Toll‐like receptor 4 on enteric glial cells, thus reducing inflammation induced by nuclear factor‐κB (NFκB) by selectively binding PPARα receptors 51. On the contrary, OEA was able to display antinociceptive properties in a PPAR‐a‐insensitive manner in mice 47.

### The endocannabinoid system and the control of gastrointestinal motility

In both animal and human GI tract, the ECs exert marked antipropulsive effects. This result is mainly mediated by the reduction in the release of acetylcholine *via* the activation of presynaptic CB1 18, 52, 53, 54. However, recent evidence suggests that along with the inhibition of acetylcholine release, the effects of the ECs on GI motility are likely to be related to the inhibition of all the components of the peristaltic reflex. In parallel with the inhibition of the release of acetylcholine, in rat models CB1 agonists were indeed able to significantly inhibit the release of both substance P and VIP, inhibiting, respectively, both the ascending contraction and the descending relaxation of the peristaltic reflex 55, 56, 57, 58. Furthermore, both the deletion of the CB1 gene 55, 56, 57 and the pharmacological blockade of these receptors 59, 60, 61 displayed prokinetic effects. Altogether, these lines of evidence seem to suggest that ECs are able to significantly reduce smooth muscle contractility, mainly by binding CB1. CB2 does not appear to play a major role in the control of intestinal motility under physiological conditions. However, studies on rodents have shown that intestinal hypermotility due to lipopolysaccharide (LPS) administration was abolished by CB2, but not by CB1 agonists 62. Hence, in animal models, CB2 agonism is more likely to inhibit intestinal motility in pathophysiological conditions associated with intestinal inflammation and immune activation.

### The endocannabinoid system and the control of visceral sensitivity

Undoubtedly, the most documented effect of the ECS is the control of visceral sensitivity and, although empirically grounded, phytocannabinoid‐based treatments have been used for centuries in a number of conditions featured by chronic pain. In recent years, several studies have elucidated the molecular mechanism by which ECs are able to reduce visceral sensation and pain. The reduction in visceral sensitivity threshold to colorectal distension was found to be dependent on both CB1 activation and CB2 activation 63, 64, 65, 66, 67. Rousseaux *et al*. have shown that after colorectal distension, orally administered probiotics were able to reduce visceral sensation in rats in a CB2‐dependent fashion 68. Moreover, in pro‐inflammatory conditions, AM124 was able to reduce the bradykinin‐induced activation of primary afferents in wild‐type but not in CB2‐deficient mice 69, further supporting the evidence that CB2 is probably involved in the control of visceral hypersensitivity in inflammatory conditions. In rodents, visceral hypersensitivity due to water avoidance stress was significantly associated with a decreased expression and function of CB1, while a reciprocal increase in TRPV1 expression was found in dorsal root ganglion (DRG) neurons 70. CB1 and TRPV1 receptors are intimately connected, and CB1 is able to inhibit TRPV1 activity either directly or indirectly through the cyclic AMP–protein kinase A 71. The treatment of DRG neurons with anandamide, whose levels are increased in psychological stress, was able to reproduce the changes in TRPV1 and CB1 expressions, while administration of CB1 agonist and/or TRPV1 receptor antagonist was able to prevent these effects 70. Furthermore, injections of corticosteroids were able to increase anandamide expression and to reproduce the reciprocal changes in the expression of CB1 and TRPV1 receptors 70. Although not completely elucidated, the mechanism underlying the reduced expression of CB1 in chronic stress conditions might rely on increased methylation of the Cnr1 gene promoter by DNMT1, which results in epigenetic modifications of CB1 expression 72. Collectively, these findings indicate that the interplay between the cannabinoid and vanilloid signalling pathways may play an important role in stress‐induced visceral hyperalgesia 73, 74. In summary, both CB1 activation and CB2 activation have been linked to the control visceral sensitivity and stress‐induced hyperalgesia in animal models. The antinociceptive effects of CB1 are probably intimately connected to a reciprocal down‐regulation of TRPV1 receptors, while CB2 is likely able to counteract the sensitizing effects of inflammatory mediators, such as bradykinin, on peripheral endings of visceral afferents.

### The endocannabinoid system and the control of intestinal inflammation

Over the past decade, many lines of evidence highlighting the role of the ECS in intestinal inflammation have been produced in both animal and pre‐clinical models 18, 75, 76. Although genetic studies failed to find any significant association between the polymorphisms in the gene encoding for FAAH and the risk of developing Crohn's disease (CD), homozygosis for the mutation Pro129Thr in FAAH gene was significantly associated with development of fistulas and extra‐intestinal manifestations in patients with CD 77. Also, in patients with ulcerative colitis (UC), the same FAAH genetic variant led to an earlier average onset of the inflammatory disease 77. Furthermore, in a recent case–control association analysis from a paediatric IBD population, the functional CB2‐R63 variant was significantly associated with the risk of developing IBDs and also linked to a more aggressive phenotype in both patients with CD and patients with UC 78. The pivotal role of the ECS in regulating intestinal inflammation has been confirmed by the evidence that both genetic ablation of FAAH and the pharmacological treatment with FAAH inhibitors prevented the development of colitis in rodents 79. In animal models, these effects are dependent on both CB1 and CB2. CB1 and CB2 agonists are indeed able to significantly reduce experimental colitis, while CB2 antagonists and CB1 knockout mice developed a more severe TNBS‐induced colitis 80, 81. Finally, it has been shown that an increase in AEA levels, induced by inhibitors of the catabolic or reuptake enzymes, significantly attenuates colitis in wild‐type mice, but not in CB1‐ and CB2‐deficient mice 82. In human beings, *ex vivo* studies have demonstrated a significantly increased expression of CB and EC levels in chronic inflammatory conditions, including IBDs, diverticulitis and coeliac disease 48, 74, 75. An overview of the reciprocal changes in CB receptors and EC level is reported in Table 1. However, both FAAH expression and levels of AEA have been reported to be decreased or increased in colitis from different studies, pointing towards the need for further studies to fully address the role of ECS in the modulation of intestinal inflammation.

**Table 1:** Reported altered expression profile of endocannabinoid system (ECS) in intestinal disease

Clinical condition		AEA	PEA	FAAH	CB1	CB2	Ref.
Ileitis	Mouse	+	=	=	+	+	54, 74
Coeliac‐like atrophy	Rat	+	+	nd	nd	nd	48
Colitis	Mouse	+/−	nd	+/−	nd	nd	82
IBD	Human	+/−	*	+/=	+/−	+	24, 62
Diverticulitis	Human	+	=	nd	=	nd	75
FD	Human	nd	nd	nd	+/*	nd	85
IBS	Human	nd	*	nd	*	+	105
NAFLD/NASH	Human	+	nd	nd	+	*	116, 117, 120, 121

+: increase; −: decrease; =: no significant change; nd: not determined; +/−: conflicting results; *: indirect evidence from administration of agonists/antagonists. IBD: inflammatory bowel disease; FD: functional dyspepsia; IBS: irritable bowel syndrome; NAFLD: non‐alcoholic fatty liver disease; NASH: non‐alcoholic steatohepatitis.

## The endocannabinoid system in gut pathophysiology

The homoeostatic role of ECS, able to regulate GI functions peripherally and centrally, represents both a blessing and a curse, making it an appealing therapeutic target and, at the same time, a challenge in selectively modulating GI functions without altering the functionality of other organs. We will now discuss in detail the evidence produced on the role of the ECS in GI disorders, namely functional dyspepsia (FD) and irritable bowel syndrome (IBS), two of the main functional gastrointestinal disorders (FGIDs), IBDs and non‐alcoholic fatty liver disease (NAFLD). We will also review the most recent advances in the possible therapeutic exploitation of manipulating ECS in the treatment of these GI disorders.

### The endocannabinoid system and functional dyspepsia

Although only few studies have investigated the potential effects of ECS in FD, there is evidence suggesting that the ECS might be an intriguing target in FD treatment, as it is involved in the modulation of some of the proposed mechanisms underlying FD pathophysiology 83, 84. In a recent study in patients with FD, Ly *et al*. have demonstrated a sustained increase in CB1 receptor availability in cerebral regions involved in the control of food intake and visceral sensitivity, suggesting for the first time a long‐term dysfunction in ECS signalling pathways in FD 85. However, whether this effect is a consequence of altered visceral sensitivity or of dysregulation in food intake still needs to be clarified. Impaired gastric accommodation, delayed gastric emptying and visceral hypersensitivity have been suggested as the underlying pathophysiological mechanisms of some FD symptoms, such as nausea, early satiety, post‐prandial fullness and pain 84, 86, 87. In experimental animals, CB receptor agonists have been shown to significantly reduce gastric emptying 88, 89. Similarly, oral administration of dronabinol (Δ9‐THC) was able to significantly reduce gastric emptying in human beings 90, 91. Furthermore, in healthy individuals, administration of a CB1 antagonist (rimonabant) was able to inhibit gastric accommodation, but not affecting gastric sensitivity, suggesting a role of ECS in the control of gastric accommodation 92. Although further studies are required to fully address the putative role of ECS in FD pathophysiology, the well‐recognized orexigenic and antiemetic effects of cannabino‐mimetics make the manipulation of ECS signalling pathway a promising strategy in FD treatment.

### The endocannabinoid system in irritable bowel syndrome

Although the pathophysiology of IBS is still not completely understood, gut motility impairment, visceral hyperalgesia, low‐grade inflammation and gut–brain axis alterations have all been associated with symptoms onset 93; hence, the ECS may represent a new therapeutic target. As ECs are known to decrease GI motility 94, 95, dronabinol, a derivative of THC, has been tested in patients affected by diarrhoea‐predominant IBS (IBS‐D) showing variable results. It has been shown that this compound was effective in decreasing the colonic transit but not colonic sensitivity, and this effect was limited to those patients carrying CB1 receptor polymorphism rs806378 96, 97. Moreover, as the activation of CB1 may reduce GI transit, the use of its antagonist may be used to increase stool frequency in constipation‐predominant IBS (IBS‐C). Actually, a selective CB1 antagonist, namely rimonabant (SR141716A), was able to increase colonic motility in mice 61. Interestingly, also the inhibition of the 2‐AG synthesizer DAGL using orlistat was found to normalize stool frequency in a mouse model of chronic constipation, without affecting basal motility 98. In addition, several lines of evidence suggested that the increase in CB1 activity might lead to a reduction in visceral sensitivity 66, 99, 100, 101. Esfandyari *et al*. have tested the efficacy of dronabinol in visceral sensitivity in a randomized, double‐blind, placebo‐controlled trial showing its ability to increase colonic compliance and relaxation *in vivo* 90. However, a further study failed to find significant difference in terms of rectal compliance between dronabinol and placebo 97. This discrepancy may be due to a different expression of CB1 in colon and rectum. Finally, several studies revealed that the ECS also participates in immune response, mainly reducing the production of inflammatory cytokines. Given the evidence for a role of low‐grade inflammation in IBS, ECs may also improve IBS symptoms by decreasing the inflammatory response 102, 103, 104. All these lines of evidence confirm that the ECS may represent a new therapeutic target in IBS; however, the risk of adverse effect still limits the use of ECs in treating FGIDs. Therefore, EC‐like compounds able to modulate ECS signalling with a good safety profile and, more importantly, without central side effects appear as promising candidates in IBS treatment. Recently, a multi‐centre randomized, double‐blind, placebo‐controlled study has shown the efficacy of orally administered PEA in decreasing the pain severity in patients with IBS. The authors found a significantly increased expression of mast cells and CB2 in IBS, while the levels of OEA were significantly reduced. Furthermore, orally administered PEA significantly improved the pain severity in these patients; however, the authors concluded that it was less obvious whether this effect was dependent on the ECS‐induced modulation of visceral hyperalgesia or on mast cell stabilization; hence, further studies evaluating the relation between ECs, inflammation and IBS are needed 105.

### The endocannabinoid system in inflammatory bowel disease

The lines of evidence showing the involvement of ECs in the regulation of inflammatory and immune response in the digestive tract inevitably promoted research on the role of ECs in IBD. The first evidence came from CB1 and CB2 knockout mice that showed a higher susceptibility to chemically induced colitis, suggesting that ECs play a key protective role against chronic inflammation 81, 106. Moreover, *in vitro* studies showed that AEA and other CB1 agonists promote wound closure in human colonic epithelium and hence might improve mucosal healing in patients with IBD 22. Furthermore, *in vitro* experiments showed that anandamide and 2‐AG increased intestinal permeability when apically administered on Caco‐2 cells, and an *in vivo* study in obese mice, a model of leaky gut, showed that the CB1 antagonist rimonabant was able to reduce plasmatic LPS level, confirming the role of ECs in regulating gut permeability 107, 108, 109. Interestingly, further studies have revealed that while CB1 mainly mediates the effects of ECs in a physiological setting, CB2 seems to assume a prevalent role during inflammatory process. Indeed, immunohistochemical studies showed that during inflammatory flares, the expression of CB2, but not of CB1, is modified and amplified 24, 62. This evidence is very intriguing as CB2 agonists may represent a new therapeutic strategy in IBD, acting directly and specifically on inflamed tissue, thus reducing central adverse effects. Finally, a protective effect of PEA has been demonstrated in human biopsies from patients with active UC, suggesting that exogenous administration of EC‐like amides may improve mucosal healing in patients with IBD 51. As NAEs are already available for treating neuropathic pain, showing a good efficacy and safety profile, further clinical trials to evaluate the therapeutic role of these compounds are clearly required.

### The endocannabinoid system in liver disease

Liver plays a major role in human homoeostasis with numerous functions, including regulation of lipid and carbohydrate metabolism, plasma protein synthesis, hormone production and detoxification. The emerging role of ECs in homoeostasis and lipid metabolism led several authors to investigate the interactions between the ECS and liver functions in normal and pathological conditions. The cannabinoid receptors are widely distributed on both hepatocytes and cholangiocytes, as on Kupffer and stellate cells, and their expression is modified during liver injury 110, 111, 112, 113. In particular, it was found that the ECS is involved in hepatic haemodynamic, cellular regeneration, liver fibrosis and lipid metabolism. As known, liver haemodynamic dysregulation plays a central role in cirrhosis, indeed portal hypertension and systemic vasodilation are involved in all major cirrhotic complications, such as ascites, variceal bleeding, liver‐related cardiomyopathy and increased risk of cardiovascular events 114, 115. Remarkably, the hypotensive effects of ECs, mainly mediated *via* CB1 activation, have been associated with cirrhosis‐induced vasodilation, and increased levels of AEA have been found in peripheral blood of patients with cirrhosis 116, 117. In rodent models of cirrhosis, the administration of CB1 antagonist was found to decrease ascites and ameliorate sodium balance, and CB1 was shown to contribute to cardiac contractility alterations related to liver cardiomyopathy, suggesting that CB1 antagonists might be used to improve cardiovascular activity in cirrhosis 118, 119. ECs have also been associated with fibrosis progression in HCV‐infected patients, suggesting a profibrotic activity of ECs. Indeed, CB1 stimulation promotes the activity of myofibroblasts and stellate cells, likely *via* an increased TGF‐β production 120, 121. On the contrary, CB2 activation seems to play a protective role against fibrosis, promoting regeneration of liver cells after acute injury. Indeed, selective CB2 agonists have been found to slow fibrosis in a rat model of cirrhosis, and CB2^−/−^ knockout mice are more sensitive to acute liver injury, showing a low regenerative response 122, 123. In summary, although ECs may worse cirrhosis progression and complications mainly *via* CB1 activation, specific CB2 agonists might slow liver fibrotic evolution.

### Endocannabinoids in non‐alcoholic fatty liver disease

The emblematic role of ECS in metabolic syndrome and obesity is already known; indeed, the CB1 antagonist rimonabant has been proposed in obesity treatment due to its beneficial effects on both bodyweight and lipid profile. However, the neuropsychiatric adverse effects have limited the clinical use of this compound. Non‐alcoholic fatty liver disease (NAFLD) and non‐alcoholic steatohepatitis (NASH) are strongly associated with metabolic syndrome, representing the ‘liver response’ to obesity, dyslipidaemia and altered carbohydrate metabolism. As ECs play a key role in liver lipid metabolism, a great interest is raised on effects of ECs on fatty liver diseases 124. Cannabinoid receptors are involved in hepatic lipogenesis, inducing specific transcriptional factors, such as SREBPs (sterol regulatory element‐binding proteins). Indeed in a mouse model with a selective deletion of hepatic CB1, a significant reduction in lipid storage during high‐fat diet has been observed 125. Intriguingly, also lipid profile and insulin resistance were improved; however, no effects on BMI have been registered in this murine model, suggesting that other mechanisms are involved in bodyweight regulation 125. Altogether, these lines of evidence support the role of ECs in hepatic steatosis and fibrotic progression, opening the possibility of new therapeutic options in treatment of NAFLD and NASH; in particular, the efficacy and safety of the CB1 antagonist rimonabant are currently under investigation in a phase III clinical trial for treatment of NASH.

## Conclusions

In the last years, accumulating lines of evidence have pointed out the homoeostatic role of the ECS in regulating intestinal motility, sensitivity and inflammation. An impairment of ECS signalling has been suggested to play a key role in several gastrointestinal disorders, such as FGIDs, IBDs and liver diseases. Even if conflicting results have been produced *in vivo*, convincing evidence suggests that pharmacological manipulation of this multifaceted system might provide new therapeutic options in treating GI diseases. The complexity and the redundancy of ECS make the manipulation of this complex system an appealing target for therapeutic purposes, although the possibility of central side effects strongly limited the current use of these compounds in clinical settings. Using peripherally acting drugs with no affinity on central cannabinoid receptors is an intriguing strategy, and as PEA formulations are already available for the treatment of chronic pain, further *in vivo* studies to test the clinical efficacy of these compounds are strongly warranted.

## Conflict of interest

The authors have no conflicting interests to declare.
